# Metabolic Interplay between the Asian Citrus Psyllid and Its *Profftella* Symbiont: An Achilles’ Heel of the Citrus Greening Insect Vector

**DOI:** 10.1371/journal.pone.0140826

**Published:** 2015-11-18

**Authors:** John S. Ramsey, Richard S. Johnson, Jason S. Hoki, Angela Kruse, Jaclyn Mahoney, Mark E. Hilf, Wayne B. Hunter, David G. Hall, Frank C. Schroeder, Michael J. MacCoss, Michelle Cilia

**Affiliations:** 1 Boyce Thompson Institute for Plant Research, Ithaca, New York, United States of America; 2 Robert W. Holley Center for Agriculture and Health, Emerging Pests and Pathogens Research Unit, USDA Agricultural Research Service, Ithaca, New York, United States of America; 3 Department of Genome Sciences, University of Washington, Seattle, Washington, United States of America; 4 Department of Chemistry and Chemical Biology, Cornell University, Ithaca, New York, United States of America; 5 Plant Pathology and Plant-Microbe Biology Section, School of Integrative Plant Science, Cornell University, Ithaca, New York, United States of America; 6 U.S. Horticultural Research Laboratory, Subtropical Plant Pathology Research Unit, USDA Agricultural Research Service, Ft. Pierce, Florida, United States of America; 7 U.S. Horticultural Research Laboratory, Subtropical Insects and Horticulture Research Unit, USDA Agricultural Research Service, Ft. Pierce, Florida, United States of America; Volcani Center, ISRAEL

## Abstract

*‘Candidatus* Liberibacter asiaticus’ (CLas), the bacterial pathogen associated with citrus greening disease, is transmitted by *Diaphorina citri*, the Asian citrus psyllid. Interactions among *D*. *citri* and its microbial endosymbionts, including ‘*Candidatus* Profftella armatura’, are likely to impact transmission of CLas. We used quantitative mass spectrometry to compare the proteomes of CLas(+) and CLas(-) populations of *D*. *citri*, and found that proteins involved in polyketide biosynthesis by the endosymbiont *Profftella* were up-regulated in CLas(+) insects. Mass spectrometry analysis of the *Profftella* polyketide diaphorin in *D*. *citri* metabolite extracts revealed the presence of a novel diaphorin-related polyketide and the ratio of these two polyketides was changed in CLas(+) insects. Insect proteins differentially expressed between CLas(+) and CLas(-) *D*. *citri* included defense and immunity proteins, proteins involved in energy storage and utilization, and proteins involved in endocytosis, cellular adhesion, and cytoskeletal remodeling which are associated with microbial invasion of host cells. Insight into the metabolic interdependence between the insect vector, its endosymbionts, and the citrus greening pathogen reveals novel opportunities for control of this disease, which is currently having a devastating impact on citrus production worldwide.

## Introduction

The phloem-limited, gram-negative, fastidious bacterium ‘*Candidatus* Liberibacter asiaticus’ (CLas) is the pathogen associated with citrus greening disease (Huanglongbing, HLB) the most serious disease of citrus worldwide [1]. CLas is transmitted by the Asian citrus psyllid (*Diaphorina citri* Kuwayama), which transmits CLas into phloem cells during feeding. Consistent with this intracellular delivery mechanism, the 1.2 Mb genome of CLas lacks type III secretion system genes, as well as extracellular degradative enzymes [2]. *D*. *citri* is a member of the Hemiptera, an order characterized by insects which transmit plant pathogens and which maintain highly co-evolved relationships with endosymbiotic bacteria. In many cases, these symbionts supplement the insect diet with metabolites which the insect is incapable of synthesizing or obtaining from its nutritionally imbalanced phloem sap diet [3]. Primary endosymbiotic bacteria are contained within the bacteriome, an interspecies organ comprised of an intimate association of insect and bacterial cells.

In addition to their function in primary metabolism, insect endosymbionts have frequently been reported to produce bioactive secondary metabolites that confer fitness advantages to their hosts. Female rove beetles of the genus *Paederus* harbor endosymbionts related to *Pseudomonas aeruginosa*, which produce the polyketide toxin pederin, conferring chemical defense to beetle larvae from predatory wolf spiders [4]. Analysis of the polyketide synthase (PKS) gene clusters and chemical structure of the isolated compound revealed pederin to be synthesized by trans-acyltransferase (*trans-*AT) type I PKS enzyme complexes. In the canonical orientation of the PKS enzyme complex, the growing polyketide is passed along a chain of synthesis and tailoring enzymes whose orientation on the genome mirrors the biochemical sequence of their action, including the AT function *in cis*. In *trans*-AT systems, polyketide extender units, typically malonyl-CoA and/or S-methylmalonyl-CoA, are loaded onto acyl carrier proteins by AT enzymes which are located in genomic regions removed from the assembly line format of the core PKS complex [5].

Metagenomics analysis of *D*. *citri* revealed the presence of a novel bacterium, ‘*Candidatus* Profftella armatura’, which is unique to *D*. *citri* and found in all populations analyzed worldwide [6]. *Profftella* has a reduced genome at 0.54 Mb, and approximately 15% of its genes are predicted to be involved in polyketide metabolism. A novel polyketide, diaphorin, was discovered in abundant quantities (up to 3 μg/500 μg insect) using HPLC mass spectrometry and was determined to be structurally related to pederin using NMR—the three additional o-methyl groups in pederin represent the primary structural difference between these two bioactive compounds [6]. Diaphorin was found to have cytotoxicity to rat B104 neuroblastoma cells and human HeLa cells at low doses in cell viability assays, but its toxicity on microbial cells has not been investigated [6]. The function of the compound in *D*. *citri*, and whether it is related to the transmission of CLas within the insect, is unknown.

We used quantitative mass spectrometry to quantify protein expression signatures associated with *D*. *citri* populations that have acquired CLas from infected citrus plants, referred to here as CLas(+), and compared these to *D*. *citri* populations fed on healthy plants, referred to as CLas(-). We identified differentially expressed proteins from both *D*. *citri* and *Profftella*, revealing patterns of changes in metabolism, defense, and immunity proteins, as well as cytosketal and vesicle trafficking proteins which may be related to cellular invasion by CLas during circulative transmission. An enhanced understanding of the *D*. *citri* proteins exploited by CLas to facilitate its transmission through the insect will inform development of specific and effective vector control treatments.

## Results

### Profftella proteins up-regulated in CLas(+) *D*. *citri*

**Fig 1:**
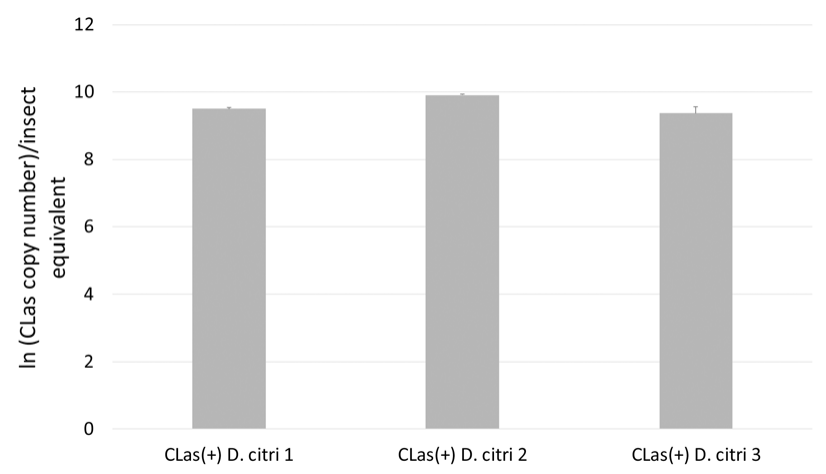
CLas qPCR analysis of *D*. *citri* samples. CLas copy number in *D*. *citri* samples (three replicates) was estimated using qPCR targeting the 16s rRNA gene. Ct values from biological samples were compared to a dilution series of a synthetic plasmid corresponding to the CLas 16s rRNA target gene. Natural logarithm of estimated CLas copy number per insect equivalent is shown on the Y-axis. All reactions were performed in triplicate (mean plus standard deviation). No signal was observed within 40 cycles for CLas(-) samples.

Quantitative PCR targeting the 16s rRNA gene of CLas was used to estimate pathogen copy number in CLas(+) samples and to confirm that no pathogen is present in CLas(-) samples (**Fig 1)** [7]. Proteins were extracted from three biological replicates of 50 insects each, collected from CLas(+) and CLas(-) colonies of *D*. *citri* reared on *C*. *sinensis* (Madam Vinous sweet orange). Proteins were extracted from flash-frozen insects and peptide samples were prepared for mass spectrometry analysis. The proteomes of CLas(+) and CLas(-) *D*. *citri* were characterized and compared using relative quantitative mass spectrometry.

**Fig 2:**
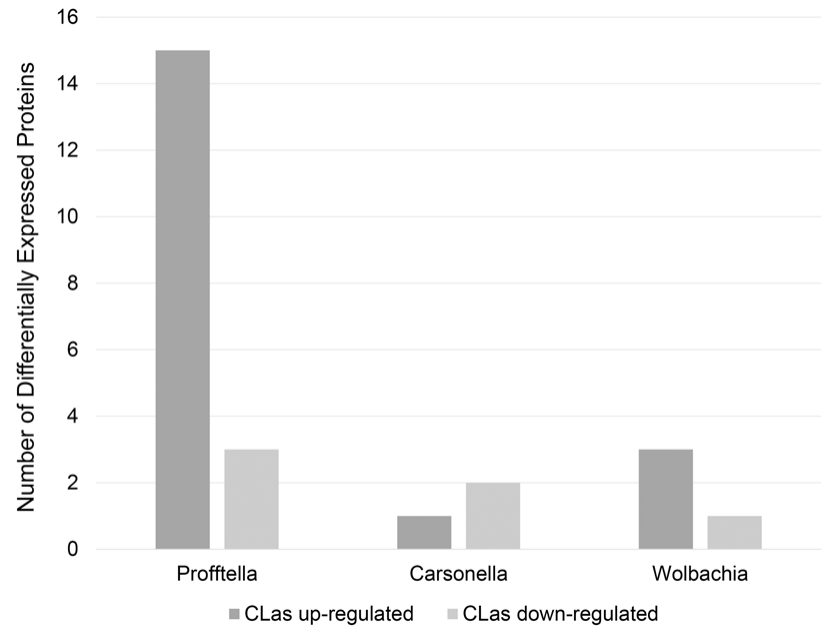
Number of *D*. *citri* endosymbiont proteins identified as differentially expressed between CLas(+) and CLas(-) samples. Out of 25 differentially expressed endosymbiont proteins, 18 were from *Profftella*, with 15 out of 18 differentially expressed *Profftella* proteins being up-regulated in CLas(+) *D*. *citri* populations.

Tandem mass spectra were searched against an in-house database of predicted proteins from *D*. *citri*, endosymbionts of *D*. *citri*, and CLas, resulting in the identification of 3764 unique proteins (**S1 Table**). Protein and peptide thresholds in Scaffold Q+ 4.4.1.1 (Proteome Software) were set at 95% probability with a minimum of 2 peptides per protein–the resulting peptide false discovery rate (FDR) was 0.09% (Scaffold peptide report: **S2 Table)**. While the majority of the proteins were *D*. *citri* proteins, proteins from bacterial endosymbionts were identified. These include 19 proteins from ‘*Candidatus* Carsonella ruddii’ DC, 44 proteins from the *Wolbachia* endosymbiont of *D*. *citri*, and 93 proteins from ‘*Candidatus* Profftella armatura’. Quantitative analysis of peptide spectra associated with each protein revealed significant differences in abundance between CLas(+) and CLas(-) *D*. *citri* whole insect samples for 340 out of 3764 total proteins (N = 3, T-test p-value < = 0.05, fold change > = 2, < = 0.5). While the majority of these were *D*. *citri* proteins, five percent of the differentially expressed proteins were from the symbiont *Profftella*. Whereas only four proteins from *Wolbachia* and three from *Carsonella* were identified as differentially expressed, 18 out of 93 *Profftella* proteins (~20%) were differentially expressed between CLas(+) and CLas(-) insects **(Fig 2)**. Similar numbers of *D*. *citri* proteins were down-regulated (160) in CLas(+) insects as were up-regulated (155); however, 15 *Profftella* proteins were up-regulated while only three were down-regulated (**S3 Table)**.

*Profftella* metabolic proteins were up-regulated in CLas(+) *D*. *citri*, including proteins predicted to be involved in polyketide metabolism (**Table 1)**. Predicted PKS loci in *Profftella* are designated as *dip* (Diaphorina pederin-like polyketide) clusters, and in many cases the *Profftella* genes have closely related orthologs in the *ped* clusters of the *Paederus* beetle symbiont system [6]. The flavin-containing monooxygenase DipN (PedG) is up-regulated by greater than four-fold in CLas(+) compared to CLas(-) *D*. *citri* samples. PedG is predicted to be involved in the oxidative cleavage of pederin from the PKS complex [4]. The insertion of the *dipN/pedG* oxygenase within the main PKS cluster results in the production of a smaller compound than is predicted by genome analysis [6].

**Table 1:** *Profftella* polyketide metabolism proteins identified from analysis of mass spectrometry data. Proteins differentially expressed between CLas(+) and CLas(-) *D*. *citri* are marked in bold (N = 3, p< = 0.05, fold difference > = 2 or < = 0.5). Average normalized peptide count from specified proteins and fold difference: CLas(+)/CLas(-).

Protein Function	Dip ID^a^	Ped ID^b^	Protein ID	p-value^c^	CLas (+)	CLas (-)	Fold Diff
**flavin-containing monooxygenase**	**DipO**	**PedG**	**AGS06825**	**0.0011**	**13.92**	**3.28**	**4.24**
**malonyl CoA-acyl carrier protein transacylase**	**DipA**	**PedD**	**AGS06838**	**0.0057**	**15.22**	**1.99**	**7.67**
**type I polyketide synthase**	**DipP**	**PedI**	**AGS06823**	**0.04**	**2.69**	**6.29**	**0.43**
methyltransferase type 11	DipM	PedE	AGS06826	0.18	21.09	16.22	1.3
acyl carrier protein	DipF	PedN	AGS06833	0.25	4.39	5.65	0.78

^a^Dip ID: *Profftella* (*Diaphorina* pederin-like polyketide) ID

^b^Ped ID: *Paederus* symbiont protein ortholog

^c^p-value from T-test comparing normalized spectral counts between CLas(+)/(-)samples

A second *Profftella* protein with a predicted role in polyketide metabolism is DipA, the ortholog of FabD from streptomycetes and PedD from the pederin system. FabD is an acyltransferase (AT) that participates in both fatty acid and polyketide biosynthesis by transferring malonyl-CoA molecules onto the acyl carrier protein component of the PKS or fatty acid synthase (FAS) module [8]. PedD is predicted to serve as a *trans-*acting acyltransferase in pederin biosynthesis, in the absence of AT domains in the PedF and PedH PKS complexes [4]. Peptide spectra matched to the *Profftella* DipA protein were observed at levels nearly eight fold greater in CLas(+) compared to CLas(-) *D*. *citri* (**Table 1)**. This function of loading extender units onto the acyl carrier protein is a critical step in polyketide assembly, although the precise nature of the diaphorin extender units is unknown, as is the substrate specificity of the AT enzymes.

One of only three *Profftella* proteins down-regulated in CLas(+) *D*. *citri* is DipP, the ortholog to the PedI protein. This large (593 kDa) protein contains a wide range of enzymatic modules, which are responsible for the initial steps of diaphorin/pederin biosynthesis, including formation of the exomethylene group. Two additional *Profftella* PKS proteins—the orthologs of the methyltransferase PedE (DipM), and the acyl carrier protein PedN (DipF)–were identified in our mass spectrometry dataset at statistically similar levels in CLas(+) and CLas(-) *D*. *citri*.

### *Diaphorina citri* proteins changes associated with CLas

Analysis of *D*. *citri* proteins identified as differentially expressed between CLas(+) and CLas(-) insects revealed a total of 155 up-regulated and 160 down-regulated proteins (**S4 Table, S5 Table)**. The up-regulated proteins include a large number of proteins involved in metabolism and cellular energy storage and utilization.

One of the most up-regulated (12-fold difference) proteins in CLas(+) *D*. *citri* relative to CLas(-) insects is enoyl-CoA hydrolase, the enzyme catalyzing the second step of fatty acid β-oxidation for the production of acetyl-CoA, which feeds into the citric acid cycle (**Table 2)**. Acyl-CoA dehydrogenase, the enzyme catalyzing the initial step of fatty acid β-oxidation, is also upregulated in CLas(+) insects. Breakdown of triglycerides leads to the production of fatty acids and glycerol. Glycerol kinase, which is also significantly up-regulated in CLas(+) *D*. *citri*, is involved in the conversion of glycerol into dihydroxyacetone phosphate, which can feed into glycolysis. The protein glycogenin, which converts glucose to glycogen, were found at seven-fold higher levels in CLas(+) *D*. *citri*, and the insect flight muscle protein titin is also up-regulated.

**Table 2:** Selected *D*. *citri* metabolism proteins upregulated in CLas(+) insects. Average normalized peptide count from specified proteins and fold difference: CLas(+)/CLas(-).

Pathway	Protein Description	Protein ID	CLas (+)	CLas (-)	Fold Diff
fatty acid β-oxidation	enoyl-CoA hydratase	XP_008485588	4.44	0.34	12.9
fatty acid β-oxidation	acyl-CoA dehydrogenase	XP_008474868	17.6	7.22	2.44
glycerol catabolism	glycerol kinase	XP_008469116	15.2	4.62	3.3
glycogen biosythesis	glycogenin-1-like	XP_008468367	10.1	1.36	7.44
glycolysis	aldose 1-epimerase-like	XP_008478895	10.5	3.7	2.84
glycolysis	phosphoglycerate mutase 1	XP_008487904	18	7.31	2.46
citric acid cycle	succinate dehydrogenase	XP_008482751	14.9	5.61	2.66
citric acid cycle	2-oxoglutarate dehydrogenase-like	XP_008483416	33.5	0	N/A
citric acid cycle	succinate-semialdehyde dehydrogenase	XP_008475373	14.9	6.64	2.24
citric acid cycle	L-2-hydroxyglutarate dehydrogenase	XP_008475179	3.04	0	N/A
valine catabolism	3-hydroxyisobutyryl-CoA hydrolase	XP_008481018	14.6	3.65	3.99
valine catabolism	propionyl-CoA carboxylase alpha chain	XP_008485697	4.4	0.98	4.47

Multiple enzymes involved in glycolysis and the citric acid cycle are upregulated in CLas(+) insects, including aldose-1-epimerase, phosphoglycerate mutase, 2-oxoglutarate dehydrogenase, and succinate dehydrogenase. The enzymes that produce the citric acid cycle intermediates succinate (succinate semialdehyde dehydrogenase) and 2-oxoglutarate (L-2-hydroxyglutarate dehydrogenase) are both significantly upregulated in CLas(+) *D*. *citri* samples. An additional class of metabolic proteins upregulated in CLas(+) *D*. *citri* are those involved in propanoate metabolism. The enzyme 3-hydroxyisobutyryl-CoA hydrolase is a component of the pathway producing propionyl-CoA from the catabolism of valine. This protein is upregulated four-fold in CLas(+) insects, as is propionyl-CoA carboxylase alpha chain, which converts propionyl-CoA into S-methylmalonyl CoA. BlastP analysis of *D*. *citri* propionyl-CoA carboxylase with the Genbank non-redundant database revealed no high homology matches to hemipteran insect proteins–the top insect match is to a termite propionyl-CoA carboxylase. Blast and Kegg analysis of propionyl-CoA carboxylase revealed that the majority of insects with sequenced genomes, including the hemipterans *Acyrthosiphon pisum*, *Myzus persicae*, *Bemesia tabaci*, and *Niaparvata lugens*, are not predicted to contain a propionyl-CoA carboxylase gene.

Insect proteins potentially involved in stress or defense responses were up-regulated in CLas(+) *D*. *citri*, including the FE4 esterase, cuticle proteins, leucine-rich repeat (LRR) domain proteins, and peritrophin, a component of the peritrophic matrix of insect intestines which is thought to protect insects from microbial invasion [9]. However, there is also evidence of down-regulation of defense and immunity proteins in CLas(+) insects (**Table 3)**. Three glutathione-S-transferases (GSTs) are down-regulated in CLas(+) *D*. *citri*–this finding is consistent with a previous study indicating significantly lower GST activity in CLas(+) insects [10]. Conflicting response of reaction oxygen species (ROS) detoxifying enzymes was observed. Separate *D*. *citri* catalase proteins were up-regulated and down-regulated in CLas(+) insects, and superoxide dismutase from *Profftella* was elevated while the same enzyme was reduced in *Carsonella*. The NF-κβ inhibitor cactus is also upregulated in CLas(+) insects, which is predicted to lead to suppression of the antimicrobial Toll signaling pathway.

**Table 3:** Selected *D*. *citri* defense and immunity proteins differentially expressed between CLas(+) and CLas(-) insects. Average normalized peptide count from specified proteins and fold difference: CLas(+)/CLas(-).

Protein Description	Protein ID	CLas (+)	CLas (-)	Fold Diff
esterase FE4-like	XP_008479062.1	28.1	12.6	2.23
larval/pupal cuticle protein H1C-like	XP_008484300.1	26.4	1.67	15.7
cuticle protein 5-like	XP_008477115.1	8.16	2.67	3.05
cuticle protein 7-like	XP_008478519.1	63.4	24.9	2.54
leucine-rich repeat transmembrane protein 2	XP_008474850.1	12.2	3.65	3.35
leucine-rich repeat-containing protein	XP_008484380.1	48.4	17.6	2.73
leucine-rich repeat-containing protein	XP_008484244.1	51.8	21	2.46
peritrophin-1-like	XP_008487571.1	5.09	0.34	14.8
catalase-like	XP_008483979.1	6.43	0	N/A
NF-kappa-B inhibitor cactus	XP_008487783.1	4.74	1.32	3.59
leucine-rich repeat-containing protein	XP_008475699.1	10.1	31.4	0.32
glutathione S-transferase-like isoform X2	XP_008475194.1	5.76	12.9	0.44
glutathione S-transferase-like isoform X2	XP_008475194.1	5.76	12.6	0.45
glutathione S-transferase omega-1-like	XP_008475955.1	0.33	2.99	0.11
catalase-like	XP_008475625.1	27.1	54.8	0.49

A large number of ubiquitin-related enzymes are differentially expressed between CLas(+) and CLas(-) insects (**Table 4)**. The ubiquitin E3 ligase is down-regulated nearly 50 fold in CLas(+) *D*. *citri*, and a 26s proteasome regulatory protein was down-regulated 25 fold. An E1 type ubiquitin activating enzyme was found to be significantly down-regulated in CLas(+) *D*. *citri*, along with other ubiquitin metabolism enzymes. In contrast, the ubiquitin thioesterase protein OTU1, a protein predicted to remove ubiquitin groups to save tagged proteins from degradation, is up-regulated in CLas(+) *D*. *citri* (**Table 4)**.

**Table 4:** *D*. *citri* ubiquitin related proteins differentially expressed between CLas(+) and CLas(-) insects. Average normalized peptide count from specified proteins and fold difference: CLas(+)/CLas(-).

Protein Description	Protein ID	CLas (+)	CLas (-)	Fold Diff^a^
ubiquitin thioesterase OTU1 isoform X1	XP_008479678.1	4.44	0.34	13
ubiquitin-conjugating enzyme E2 G2-like	XP_008471000.1	5.76	0.97	5.89
26S proteasome non-ATPase regulatory subunit 3	XP_008484303.1	0.33	8.68	0.03
small ubiquitin-related modifier 1	XP_008471879.1	1.71	6.66	0.25
ubiquitin carboxyl-terminal hydrolase 7	XP_008484874.1	5.44	16.3	0.33
E3 ubiquitin-protein ligase Bre1-like	XP_008478774.1	0.33	16.8	0.01
ubiquitin-like modifier-activating enzyme 5	XP_008480018.1	2.38	6.64	0.35

An additional class of relevant down-regulated proteins in CLas(+) insects are those proteins involved with cytoskeletal remodeling and cell-cell communication (**Table 5)**. These include proteins predicted to be involved in endocytosis, such as the Rab GTPase Rab5c, and coatomer, a protein complex coating membrane-bound vesicles. Alpha catenin, ankyrin, and nesprin are examples of *D*. *citri* cytoskeletal related proteins significantly downregulated in CLas (+) insects. Two zonadhesin-like proteins, which are predicted to mediate adhesion between cells in the extracellular matrix, are down-regulated in CLas(+) *D*. *citri*, as is proteoglycan 4, a heavily glycosylated protein component of the extracellular matrix.

**Table 5:** Selected *D*. *citri* proteins associated with cell adhesion, endocytosis, and the cytoskeleton downregulated in CLas(+) insects. Average normalized peptide count from specified proteins and fold difference: CLas(+)/CLas(-).

Protein Description	Protein ID	CLas (+)	CLas (-)	Fold Diff
ras-related protein Rab-5C	XP_008474145.1	3.71	12.2	0.3
coatomer subunit beta-like	XP_008486523.1	1.69	4.31	0.39
alpha catenin	XP_008486042.1	2.02	9.96	0.2
nesprin-1-like	XP_008475701.1	6.05	23.5	0.25
nesprin-1-like	XP_008475700.1	14.5	39.7	0.36
nesprin-3-like	XP_008480932.1	0	4.31	0
ankyrin-3-like	XP_008470928.1	1.34	3.64	0.37
zonadhesin-like	XP_008477666.1	0.33	7.32	0.04
zonadhesin-like	XP_008483063.1	1.69	11.6	0.14
proteoglycan 4-like	XP_008488247.1	19.3	53.7	0.36

### Proteome analysis of Percoll gradient fractions enriched for CLas and endosymbionts

Proteome analysis of whole insects was complemented by parallel analysis of enriched samples of microbial cells and associated insect cells fractionated from homogenates of CLas(+) and CLas(-) *D*. *citri* samples using Percoll gradient centrifugation. Out of a total of 32 proteins found to be up-regulated in CLas(+) Percoll gradient samples, 12 (37%) are *Profftella* proteins while the remaining 20 are *D*. *citri* proteins (**S6 Table**). *Profftella* proteins upregulated in CLas(+) Percoll fractions include the DipM/PedE methyltransferase involved in polyketide biosynthesis. None of the 12 proteins found to be down-regulated in CLas(+) Percoll gradient samples were derived from *Profftella* (**S7 Table**). In addition to 76 *Profftella* proteins identified from Percoll gradient analysis, 22 *Wolbachia* and 9 *Carsonella* proteins were identified, none of which were differentially expressed between CLas(+) and CLas(-) samples [**S8 Table** (protein report)**, S9 Table** (peptide report)].

### HPLC-mass spectrometry analysis of *D*. *citri* polyketides

**Fig 3:**
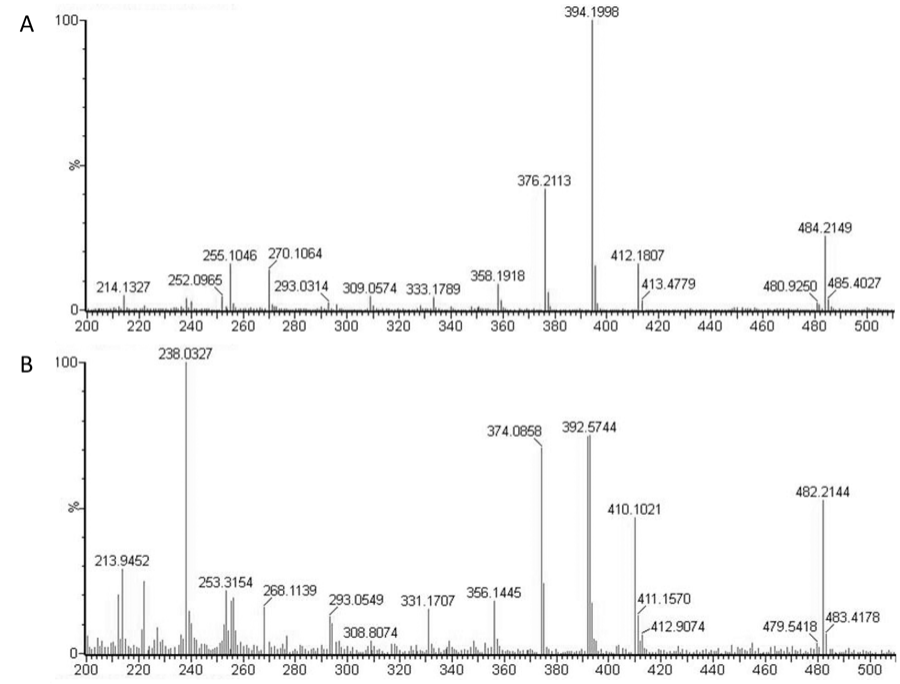
Fragmentation pattern of diaphorin and the diaphorin-related compound.

**Fig 4:**
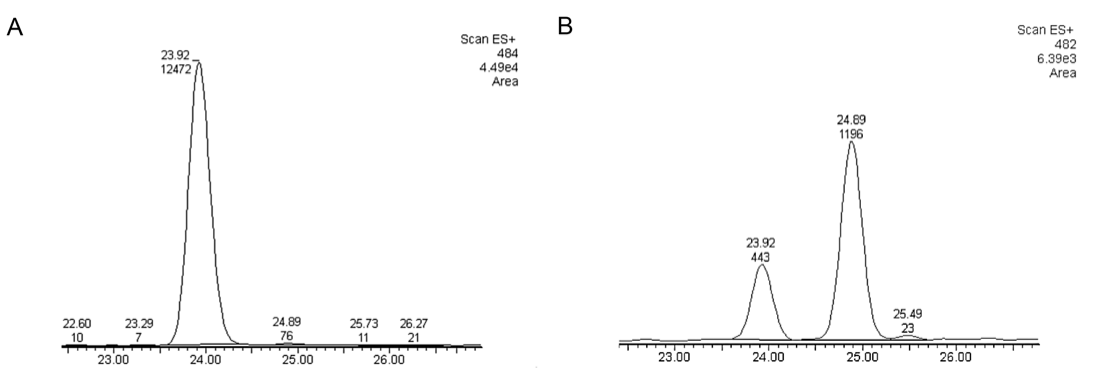
Extracted ion chromatograms from HPLC-MS analysis of CLas(+) and CLas(-) *D*. *citri* methanol extracts. A) diaphorin (484 *m/z*) and B) novel predicted polyketide (482 *m/z*). Retention time (minutes) on the X axis, signal intensity on the Y axis with peak area integration indicated above spectral peaks.

**Fig 5:**
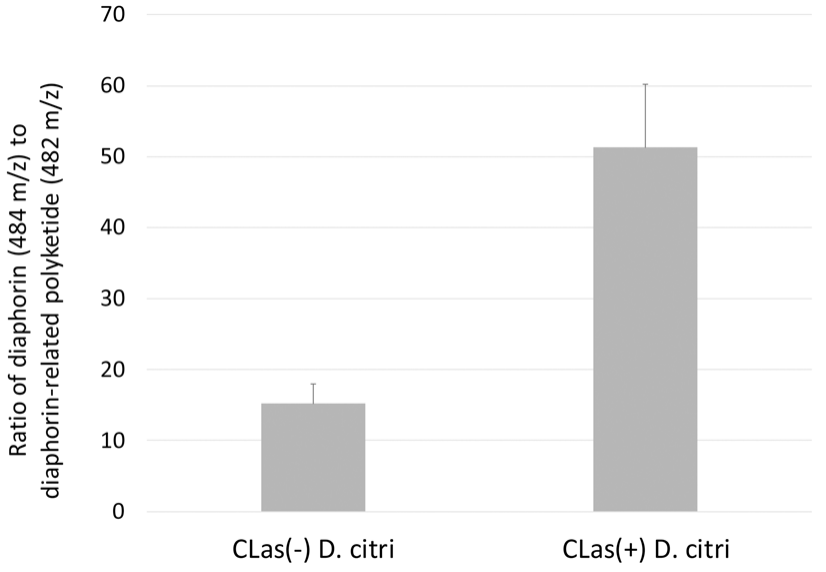
Ratio of integrated peak area from extracted ion chromatograms of 484 m/z and 482 m/z compounds from HPLC-MS analysis of CLas(+)/(-) *D*. *citri* methanol extracts. CLas(-): N = 5; CLas(+): N = 6. Mean ratio of 484/482 plus standard error given for CLas(+)/(-) samples.

Metabolite analysis of methanol extracts from CLas(+) and CLas(-) insects was performed to quantify levels of the diaphorin polyketide. Methanol extracts were prepared from 50 insects each from colonies of CLas(+) and CLas(-) *D*. *citri*. At the same time, DNA was extracted from 10 insects each collected from the same colonies and used for qPCR analysis of *Profftella* copy number. Polyketide extraction from *D*. *citri* was carried out following, with some modifications, the method used in the initial discovery of diaphorin [6]. Dried methanol extracts were reconstituted in isopropanol at a concentration of 1 mg/mL–these solutions were used for high pressure liquid chromatography-mass spectrometry (HPLC-MS) analysis of diaphorin. HPLC-MS analysis revealed a large peak containing a compound with a mass/charge ratio (*m/z*) of 484.2, which was the previous *m/z* reported for diaphorin [6]. A smaller peak was observed at a retention time approximately one minute later than diaphorin (on a total gradient run time of 62 minutes), which contained a compound with *m/z* 482.2. Both peaks displayed strong absorbance at 200 nm, consistent with absorbance spectroscopy data reported for diaphorin, and in-source fragmentation revealed the two compounds to have similar fragmentation patterns (**Fig 3**). Extracted ion chromatograms for both compounds revealed that the 482 *m/z* compound was incompletely resolved by HPLC, and that a small amount co-elutes with the 484 *m/z* compound (**Fig 4**). Quantitative analysis of several biological replicates of *D*. *citri* methanol extracts revealed a significant increase in the ratio of diaphorin to the diaphorin-related polyketide in CLas(+) insects (**Fig 5**).

### *Profftella* copy number in CLas(+) and CLas(-) *D*. *citri*

qPCR with primers targeting the *Profftella* 16s rRNA gene was performed on insects from the same samples as were used in the metabolite analysis experiment described above. DNA was extracted from adult *D*. *citri* and used for quantitation of *Profftella* copy number in SYBR green qPCR experiments using published primers [11]. No significant difference was found in *Profftella* copy number between CLas(+) and CLas(-) *D*. *citri* (**S1 Fig**).

## Discussion

Understanding the factors enabling successful transmission of ‘*Candidatus* Liberibacter asiaticus’ (CLas) by the Asian citrus psyllid (*Diaphorina citri*) is critical to advancing effective citrus greening disease control strategies. The central question we sought to address with this research is as follows: What cellular and metabolic changes in the insect vector are associated with acquisition and transmission of the plant pathogen? Fluorescence *in situ* hybridization with confocal microscopy has revealed the distribution of CLas in several *D*. *citri* organs and tissues, including the haemolymph, midgut, and salivary glands [12]. Circulative transmission of CLas requires that the bacteria cross cellular boundaries in the psyllid digestive tract and salivary gland to be injected into a healthy plant as a salivary component. Coordinated down-regulation of *D*. *citri* proteins involved in cell-cell interaction, endocytosis, vesicle trafficking, and the cytoskeleton was observed in CLas(+) insects. The down-regulation of these proteins may indicate that changes in cell-cell interactions, membrane trafficking, and cytoskeletal configuration are induced by CLas to facilitate its circulative transmission within the host insect, or these changes may represent cellular modifications made by the host as part of a defense response against the invading pathogen.

The systemic invasion of a range of *D*. *citri* organs by CLas suggests that this plant pathogen should also be considered an insect pathogen. In the trigger mechanism of host cell invasion, bacteria inject effector proteins into a host cell using a type III secretion system (T3SS), resulting in changes in the host cell leading to bacterial engulfment and internalization [13]. As the CLas genome lacks components of the type III secretion system, invasion of insect cells by the bacterium is likely to occur by a zipper mechanism, involving endocytosis mediated by interactions between bacterial outer membrane proteins and proteins on the host cell surface. Internalization of *Listeria monocytogenes* by mammalian cells proceeds through a zipper mechanism, wherein membrane and cytoskeleton rearrangements in host cells lead to bacterial internalization [13]. It is likely that CLas exploits conserved cellular machinery within the insect to facilitate its transmission, and the above-mentioned protein changes may be induced by CLas virulence factors as part of the pathogen’s cellular invasion strategy. Alternatively, *D*. *citri* may be downregulating proteins involved in cellular adhesion and endocytosis in an effort to prevent CLas from hijacking these systems for its own benefit. Intracellular pathogens have been reported to hijack the host ubiquitin system to manipulate the host cell and facilitate invasion [14]. Down-regulation of ubiquitin proteins and the 26s proteasome regulatory factor may reflect manipulation of the vector proteolysis system by CLas, perhaps to prevent the targeting of pathogen proteins for degradation.

Although no comparable study has been published for the *D*. *citri*/CLas system, the fitness of the potato psyllid (*Bactericera cockerelli*) has been found to be negatively affected by infection with the related ‘*Candidatus* Liberibacter solanacearum’, which is associated with Zebra chip disease in potato [15]. The up-regulation of *D*. *citri* proteins such as FE4 esterase, peritrophin, and leucine rich repeat domain proteins in response to CLas is consistent with an insect stress response to the citrus greening pathogen. However, other responses to CLas, such as upregulation of the NF-κβ inhibitor cactus and downregulation of lysozyme and glutathione-S-transferases, suggest that *D*. *citri* defense responses may be suppressed, enabling circulation of the citrus greening pathogen through the insect. Similar to other Hemiptera, the repertoire of antimicrobial defenses is reduced in *D*. *citri* relative to other insects. The need to accommodate their colonization by beneficial symbionts has been invoked to explain the lack of the Immune Deficiency (IMD) pathway and antimicrobial peptide genes in Hemipteran genomes [16]. In our experimental system, CLas(+) insects are impacted directly by the presence of the citrus greening bacterium in their bodies, and indirectly by feeding on infected citrus plants, which may be mounting a defense response characterized by the production of reactive oxygen species or other physiological changes affecting plant quality. The indirect effects on the insect of changes in nutritional composition of CLas infected plants may contribute to the observed proteome changes in CLas(+) insects. Within a population of *D*. *citri* feeding on CLas infected plants, some insects do not acquire the pathogen, others acquire the pathogen but are not capable of transmission, and only a small percentage (<12%) are capable of transmitting the pathogen [17]. Understanding why some individual insects feed on infected plants and never become competent to transmit the pathogen may illuminate details of CLas transmission barriers within the insect.

Metabolic changes in *D*. *citri* associated with CLas may be the result of manipulation of the vector by the citrus greening pathogen to ensure its survival and dispersal. The induction of fatty acid β-oxidation in conjunction with increased glycogen synthesis suggests that central changes in the insect’s metabolism related to energy storage and utilization are associated with feeding on CLas(+) plants and acquiring and harboring the citrus greening pathogen. The coordinated up-regulation of these enzymes suggests that metabolic changes in *D*. *citri* associated with CLas include breakdown of triglycerides and metabolism of the breakdown products into compounds which can enter glycolysis or the citric acid cycle for energy production. Glycogen, a complex polysaccharide, is an important energy reserve in insects which can be mobilized more rapidly than triglycerides. Several recent studies have illustrated how plant viruses can directly and indirectly manipulate their host and vector to ensure their own survival and dispersal. The behavior and physiology of vectors of circulative viruses are influenced by the infection status of the plant in several ways, including in responses to visual and olfactory cues, in physiological and nutritional changes, and apparently also by changes in the vector once they have acquired virus. Mechanistic studies have recently shown that viruliferous insects spend more time feeding and salivating in the phloem than non-viruliferous insects, which would also help increase the chance of virus inoculation [18]. *D*. *citri* adults prefer CLas infected plants initially, but after feeding for a period of time they prefer to settle on healthy plants [19]. Infection with CLas also impacts the production of plant volatiles and metabolites [19, 20]. All of the above mentioned findings contribute to an enhanced probability of transmission and survival of the pathogen, and this has led to the proposal of the “Vector Manipulation Hypothesis” [21]. The up-regulation of the *D*. *citri* flight muscle protein titin suggests that changes in insect physiology potentially affecting flight may be associated with CLas. Behavioral studies are needed to determine whether the changes in metabolic enzymes observed in CLas(+) *D*. *citri* have an impact on host plant choice, feeding patterns, and flight characteristics.

Several polyketide biosynthesis proteins from the *D*. *citri* endosybiont *Profftella* were identified in protein samples extracted from whole insects and microbe-enriched Percoll gradient fractions. Electron microscopy and fluorescence *in situ* hybridization reveal that *Profftella* cells are contained in a syncytial cytoplasm within the *D*. *citri* bacteriome, while *Carsonella* cells are found on the perimeter encircling the bacteriome [6]. Both *Profftella* and *Carsonella* have highly reduced genomes, and the physical relationship between these endosymbionts and the host insect create an environment amenable to shared metabolic pathways, where one organism can complement the metabolic capacities of another. *Profftella* is predicted to be a polyketide synthesis specialist based on its gene content, and there are high basal levels of polyketide synthase and acyl carrier proteins constitutively present. CLas(+) insects were found to have dramatically elevated levels of two proteins involved in polyketide biosynthesis, one catalyzing the transfer of activated extender units onto acyl carrier protein for incorporation into the growing polyketide chain (DipA), and the other responsible for oxidative cleavage and release of the nascent polyketide (DipO). In contrast, the protein DipP, a large multi-subunit enzyme complex responsible for initiating diaphorin biosynthesis, is down-regulated in CLas(+) *D*. *citri*. Supply of extender units by DipA and cleavage of the nearly complete (save for final O-methyltransferase tailoring) polyketide by DipO may act to regulate diaphorin production more effectively than by increasing production of large enzyme complexes such as DipP. Regulation of *Profftella* polyketide metabolism is poorly understood, but an increase in supply of polyketide precursors, which are loaded onto the acyl carrier protein by DipA, may be a factor which stimulates production of diaphorin.

During our mass spectrometry analysis of diaphorin in *D*. citri, we discovered a compound predicted based on its fragmentation pattern to be a diaphorin-related polyketide. The ratio between levels of diaphorin and this novel related polyketide is significantly increased in CLas(+) compared to CLas(-) *D*. *citri*, suggesting changes in *Profftella* polyketide metabolism in response to the presence of the pathogen or in direct or indirect response to changes induced by the pathogen in infected plants. The up-regulation of PKS proteins in CLas(+) *D*. *citri* may be a specific response of *Profftella* to the presence of CLas, as part of an infection response that may be mediated by *D*. *citri*. More studies are needed to establish the natural variation of both *Profftella* copy number and diaphorin concentration in *D*. *citri* populations, rigorously accounting for variation in insects and host plants in addition to the presence of absence of CLas.

Given the metabolic interdependence commonly ascribed to relationships between hemipteran insects and their microbial endosymbionts, we hypothesized that the insect host may play a role in regulating production of diaphorin by *Profftella* by supplying the symbiont with the extender units used as polyketide building blocks. The most common extender units for type I polyketide biosynthesis are malonyl-CoA and S-methylmalonyl-CoA [22]. *D*. *citri* is unusual among insects in that it is predicted to have the genetic capacity to make S-methylmalonyl-CoA from propionyl-CoA by means of propionyl-CoA carboxylase. The predicted *D*. *citri* propionyl-CoA alpha chain protein is much shorter than its orthologs in other animals (79 amino acids compared to 300–750 amino acids), raising the question of whether its metabolic function is conserved. The *D*. *citri* protein encompasses a predicted ATP Grasp domain, which is shared by several critical metabolic enzymes catalyzing the reaction between a carboxylic acid and a nucleophile [23]. Propionyl-CoA carboxylase is a central metabolic enzyme in many animals, with the S-methylmalonyl-CoA produced in this reaction being further metabolized into succinyl-CoA for entry into the citric acid cycle. Vitamin B12 is a cofactor required for the synthesis of succinyl-CoA by methylmalonyl-CoA mutase; no detectable levels of vitamin B12 have been observed in many diverse insect species, and this cofactor is not an essential requirement in insect diets [24]. Termites are exceptional among insects in that they have been reported to have high levels of vitamin B12, presumably synthesized by endosymbiotic microorganisms, which may have led to the retention of the complete metabolic pathway for interconversion of propionyl-CoA to succinyl-CoA [24]. Neither *D*. *citri* nor its endosymbionts is predicted to have the genes required for further metabolism of S-methylmalonyl-CoA into succinyl-CoA.

**Fig 6:**
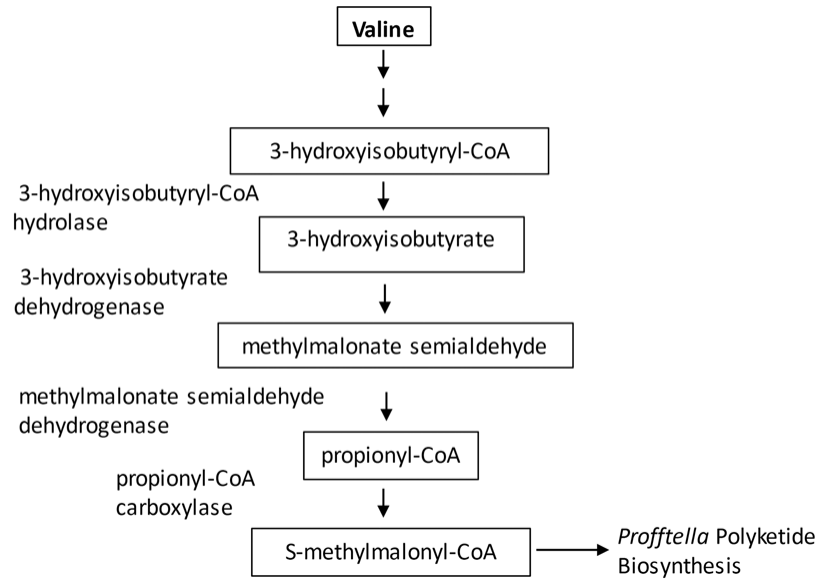
Metabolic interplay between *D*. *citri* and *Profftella*. Proposed model of shared metabolic pathway wherein S-methylmalonyl-CoA from *D*. *citri* valine catabolism is provisioned to *Profftella* for polyketide biosynthesis.

Peptides derived from propionyl-CoA carboxylase alpha chain were more abundant in CLas(+) than CLas(-) *D*. *citri* protein samples. The protein 3-hydroxyisobutyryl-CoA hydrolase, involved in the formation of propionyl CoA from the catabolism of valine, was also significantly up-regulated in CLas(+) insects. Additional *D*. *citri* valine catabolism proteins identified are 3-hydroxyisobutyrate dehydrogenase and methylmalonate semialdehyde dehydrogenase, with the latter found to be expressed at high levels in Percoll fraction and whole insect samples. Pathway analysis suggests that S-methylmalonyl-CoA may be produced in the psyllid bacteriome through catabolism of valine, and provisioned to *Profftella* for use as a polyketide building block. This model predicts that as a component of the response of *D*. *citri* to CLas, valine catabolism proteins are up-regulated to provide S-methylmalonyl-CoA to *Profftella* for diaphorin production (**Fig 6)**. Valine used by *D*. *citri* for production of S-methylmalonyl-CoA may be obtained from the insect diet, or alternatively it may be obtained from *Carsonella*, which has an intact valine biosynthesis pathway. Neither *Carsonella* nor *Profftella* is predicted based on gene content to have valine catabolic activity. While the structure of diaphorin supports the model of S-methylmalonyl-CoA as the polyketide extender unit, it is also possible that malonyl-CoA is incorporated into the growing polyketide chain, followed by C-methylation with a methyltransferase embedded in one of the PKS complexes–additional biochemical studies are needed to discriminate between these two hypotheses.

There are many unanswered questions about the function of *Profftella* and diaphorin in relation to the metabolism and physiology of *D*. *citri* and the transmission of CLas. More detailed understanding of the biosynthesis of diaphorin and the nature of its biological targets will illuminate the mechanisms of this unique defensive symbiosis. Interactions between *D*. *citri* and its endosymbionts are highly specific, representing promising targets for vector control strategies aimed at curtailing the spread of the citrus greening pathogen.

## Materials and Methods

### Insect Colonies

All *D*. *citri* DNA, protein, and metabolite samples were obtained from mixed sex adult *D*. *citri* reared on either healthy or CLas infected ‘Madam Vinous’ sweet orange [*Citrus sinensis*].

### Percoll density gradient centrifugation

A Percoll density gradient centrifugation method used to purify a phytoplasma from lettuce was modified and used to purify enriched microbial fractions from homogenized *D*. *citri* samples [25]. One hundred and fifty freshly collected (not frozen) *D*. *citri* were homogenized in 10 mL CLas isolation buffer (100 mM Sodium Phosphate pH 7.2, 5 mM dithiotreitol, 1% polyvinylpyrrolidone, 5% sucrose, 5 mM EDTA) using a Brinkman homogenizer. The insect homogenate was filtered using a 40 micron Millipore vacuum filter. Filtered samples were pelleted by centrifuging for 30 minutes at 15,500 RPM (41,000 x g), 4°C using an SW-32Ti rotor. The supernatant was discarded and the pelleted cells were resuspended in 2 mL of CLas suspension buffer (100 mM sodium phosphate pH 7.2, 5 mM dithiotreitol, 5% sucrose, 5 mM EDTA).

The Percoll gradient was prepared in 1” x 3.5” thin-walled ultracentrifuge tubes (Beckman Coulter). The Percoll stock solution was made by mixing 9 volumes of Percoll with 1 volume of 2.5 M sucrose in water. More dilute solutions of Percoll were made by mixing the stock solution with sucrose phosphate buffer (0.25 M sucrose, 10 mM sodium phosphate pH 7.0). The first layer was a 60% Percoll solution (6 mL) on the bottom of the tube. The second (11 mL of 30% Percoll) and third (11 mL 15% Percoll) layers were slowly dripped alongside the wall of the tube to layer. The filtered and concentrated cell pellet was then loaded onto the top of the gradient. Centrifuge tubes were balanced and samples were centrifuged for 25 minutes at 14,800 RPM (37,000 x g) at 4°C with a chilled SW-32Ti rotor. Following centrifugation, the gradient was separated into six fractions (five mL each) which were removed sequentially by pipetting from the top down. Bands were observed in fractions two and three, which were analyzed by qPCR and mass spectrometry. The fractions were washed twice with 10 mL of 0.85% NaCl. The salt solution was added, tubes were inverted to mix/wash and centrifuged for 10 minutes at 4°C, 3500 rpm, and the supernatant was removed by pipetting and discarded. After the second wash, 500 μL of 0.85% NaCl was left in the tube and used to resuspend the purified cells.

### *Profftella* and *Liberibacter* qPCR

Genomic DNA samples were isolated from whole insect and Percoll fraction samples using the Qiagen DNeasy® Blood &Tissue Kit. For whole insect samples, 10 adult *D*. *citri* were collected, flash frozen, and cryoground (Retsch Mixer Mill MM 400) in 2 mL microcentrifuge tubes containing three 3.2 mM steel balls. The Qiagen Supplementary Protocol for Purification of total DNA from insects using the DNeasy® Blood &Tissue Kit was followed, beginning with addition of Buffer ALT to the ground insect powder. The Qiagen Protocol for Purification of Total DNA from Animal Blood or Cells was followed to purify DNA from 50 μL of Percoll fraction cells using the DNeasy® Blood &Tissue Kit. The Eppendorf centrifuge rotor F45-24-11 was used for all purifications with maximum speed of 13,200 RPM (16,100 x g).

All qPCR reactions were performed on an Applied Biosystems 7900 HT Fast Real-Time PCR system. For CLas analysis, Taqman qPCR assays were performed using probe and primers targeting the CLas 16s rRNA gene–published primer and probe sequences were used, substituting a FAM/ZEN/IBFQ dye/quencher probe for the FAM/TAMRA probe [7]. Primers and probes were synthesized by Integrated DNA Technologies (Coralville, IA). Taqman Universal PCR Master Mix (Life Technologies, Foster City, CA) was used for 20 μL qPCR reactions containing forward and reverse primer (600 nM each), probe (300 nM) and one μL of purified DNA. Thermocycler protocol was as follows: ten minutes at 95°C; 40 cycles of 15 seconds at 95°C, 60 seconds at 60°C. Samples that did not come to threshold at the completion of the run were scored as Ct = 40. For absolute quantitation of CLas copy number, a DNA fragment containing the target region of the 16s rRNA gene was synthesized and cloned into the pUC57 plasmid (Genscript, Piscataway, NJ). Dilution series of this plasmid standard were prepared and used in qPCR reactions to establish a standard curve allowing conversion of Ct values from sample data into an estimate of the CLas genome copy number. Standard curve for whole *D*. *citri* qPCR analysis: [Ct value = -1.492ln(x) + 42.872], where x = estimated CLas copy number.

For *Profftella* analysis, Sybr Green qPCR reactions were run using published primers targeting the 16s rRNA gene [11]. Fast SYBR green Master Mix (Life Technologies) was used in 20 μL reactions containing 250 nM each forward and reverse primer and one μL of purified DNA. DNA concentration was measured using a Nanodrop (Thermo Scientific, Wilmington, DE) and adjusted such that each qPCR reaction used 250 ng DNA starting material. Thermocycler protocol was as follows: 95°C for 20 seconds; 40 cycles of three seconds at 95°C, 30 seconds at 60°C; dissociation curve of 15 seconds at 95°C, 15 seconds at 60°C, 15 seconds at 95°C (final ramp rate 2%). Dilution series of *Profftella* 16s synthetic plasmid was used for absolute quantitation as described for CLas above. Standard curve for *Profftella* qPCR analysis: [Ct value = -1.39ln(x) + 40.467], where x = estimated *Profftella* copy number.

### *D*. *citri* protein extraction and quantitation

*D*. *citri* proteins were precipitated and prepared for mass spectrometry analysis following methods modified from [26]. For whole insect protein extraction, adult *D*. *citri* (20–40 mg each sample) were flash frozen and cryoground in 2 mL microcentrifuge tubes containing three 3.2 mM steel balls. Protein precipitation solvent (10% trichloroacetic acid in acetone with 2% beta-mercaptoethanol) was made fresh and kept on ice until use. Precipitation solvent (500 μL) was added to ground psyllid powder, the metal beads were removed using forceps, and the sample was agitated using a vortexer and placed in the -20°C freezer for five hours to precipitate. Precipitated protein pellets were washed three times with one mL ice-cold acetone, and after decanting the supernatant from the final wash the pellet was dried to completion. Pellets were resuspended in 200 μL protein reconstitution solvent [8 M urea, 50 mM triethylammonium bicarbonate (TEAB) in water]. Pellets were reconstituted overnight with shaking. Samples were centrifuged at full speed to pellet insoluble material, and the supernatant was collected for protein analysis.

For Percoll gradient fraction protein extraction, one mL precipitation solvent was added to purified cells and the sample was sonicated to rupture cells. Several rounds of sonication (30 seconds, 15% amplitude, Branson digital probe sonicator, Danbury, CT) were needed to fully disrupt pellet, with samples chilled on ice between rounds of sonication to avoid heating. Samples were placed in the -20°C freezer for overnight precipitation, and washing and reconstitution was performed as for whole insect protein samples.

Quick Start Bradford Protein Assay (Bio-Rad, Hercules, CA) was used to determine concentration of protein samples. Gel electrophoresis was used as a quality control check to validate the Bradford results. Ten μg of psyllid protein sample was adjusted to a volume of 25 microliters using phosphate buffered saline solution. 25 microliters of 2x Laemmli Sample Buffer (Bio-Rad) containing 5% beta-mercaptoethanol was added to each protein sample. Samples were incubated at 70°C for 10 minutes and run on a 10% Mini-PROTEAN TGX Pre-cast gel (Bio-Rad) at 80V for 2 hours, using SDS-PAGE running buffer and a Precision Plus Protein Kaleidoscope standard (Bio-Rad).

After running, the gels were removed from their casing and transferred to a plastic container to stain using Invitrogen NOVEX Colloidal Blue Staining Kit, following instructions for Tris-Glycine gels (Life Technologies). Gels were stained overnight on a rocker. To de-stain, the gels were rocked in Milli-Q water for a few hours, replacing the water as needed. Once de-stained, gels were scanned and samples were then compared. Bradford Assays were repeated for samples that showed noticeably darker or lighter bands in the gel.

### Peptide sample preparation for mass spectrometry analysis

For each whole insect sample, 200 μg of protein was prepared for mass spectrometry analysis, and 80 μg of protein was prepared from Percoll gradient fractions. For reduction, a 100 μL solution of each protein in 10 mM dithiotreitol, 50 mM TEAB was prepared, and samples were vortexed, centrifuged briefly, and incubated for one hour at 30°C. After cooling samples to room temperature, methyl methanethiosulfonate (MMTS) in 50 mM TEAB was added to reach a final concentration of 30 mM MMTS, samples were vortexed, centrifuged, and incubated for one hour at room temperature in the dark. Samples were diluted with TEAB if necessary to reduce concentration of urea below 1 M. Sequencing grade, modified trypsin (Promega, Madison, WI) was added to protein samples (target ratio of trypsin: protein is between 1:20–1:100 by weight). Samples were vortexed, centrifuged, and incubated at 30°C overnight.

Oasis MCX solid phase extraction cartridges, 30 μM particle size, (Waters, Milford, MA) were used for peptide clean-up. Trypsin-digested peptide samples were dried in a vacuum concentrator and reconstituted in 0.1% formic acid in water. Concentrated formic acid (5 μL) was added to peptide samples, which were spotted on litmus paper to confirm pH>3. Cartridges were conditioned with 1) 1 mL 100% methanol, 2) 1 mL 3% ammonium hydroxide in water, 3) 2 mL 100% methanol, 4) 3 mL 0.1% formic acid in water. Samples were added to the column, salts were washed with 1 mL 0.1% formic acid in water, neutrals were washed with 1 mL 0.1% formic acid in methanol, and peptides were eluted in 1 mL 3% ammonium hydroxide in methanol and dried down in a speed-vac.

### Peptide mass spectrometry

The dried tryptic digests were solubilized in 600 ul of 0.1% trifluoracetic acid and 2% acetonitrile by vortexing for 10 minutes at 37 C and bath sonication for 5 minutes to give a final concentration of 0.333 ug/ul, based on prior BCA protein assays. The solubilized digests were centrifuged at 10,000 g for 5 minutes to pellet any particulates that might cause HPLC clogging, and the a portion of each supernatant was carefully removed and placed into autosampler vials.

All mass spectrometry was performed on an LTQ-Orbitrap-Fusion (Thermo Fisher Scientific). Three microliters (~1 ug) was loaded from the autosampler onto a 150-μm Kasil fritted trap packed with Jupiter C12 90 Å material (Phenomenex, Torrance, CA) to a bed length of 2 cm at a flow rate of 2 ul/min for five minutes. After loading and desalting, the trap was brought in-line with a pulled fused-silica capillary tip (75-μm i.d.) packed with 30 cm of Reprosil-Pur C18-AQ (3-μm bead diameter, Dr. Maisch GmbH, Ammerbuch-Entringen, Germany) mounted in an in-house constructed microspray source. Peptides were eluted off the trap and column using a Waters Nanoacquity binary UPLC pump using a gradient of 2–25% acetonitrile in 0.1% formic acid over 100 minutes, followed by an additional 25–60% gradient over 40 minutes. The trap and column were subsequently washed for five minutes each with 60% and 95% acetonitrile in 0.1% formic acid, all at a flow rate of 250 nL/min.

The mass spectrometer was operated using electrospray ionization (2 kV) with the heated transfer tube at 275 C using data dependent acquisition (DDA) in “Top Speed” mode, whereby one orbitrap mass spectrum (*m/z* 400–1600 with quadrupole isolation) was acquired with multiple linear ion trap tandem mass spectra every three seconds or less. The resolution for MS in the orbitrap was 120,000 at *m/z* 200, and for MS/MS the linear ion trap provided unit resolution. The automatic gain control target for MS in the orbitrap was 2e^5^, whereas for MS/MS it was 1e^4^, and the maximum fill times were 20 and 35 msec, respectively. The MS/MS spectra were acquired using quadrupole isolation with an isolation width of 1.6 *m/z* and HCD normalized collision energy of 30%. The precursor ion threshold intensity was set to 1e^4^ to trigger an MS/MS acquisition. Furthermore, MS/MS acquisitions were allowed for precursor charge states of 2–7. Dynamic exclusion (including all isotope peaks) was set for 30 seconds using monoisotopic precursor selection with a mass error of 15 parts per million (ppm). The fragment ions were analyzed in the linear trap using the “rapid” scan rate. Each sample was analyzed in triplicate, where the choice of precursor selected for MS/MS was varied for each replicate as follows–most intense precursor, lowest charge state sorted from highest *m/z* to lowest *m/z*, or lowest *m/z*. Use of three different decisions for each triplicate was an attempt to capture a larger number of precursors for each sample.

### Mass spectrometry data analysis

Mass spectrometry data files were searched against a combined database of predicted *D*. *citri*, endosymbiont (*Carsonella*, *Profftella*, and *Wolbachia)*, and CLas proteins using Mascot Daemon 2.3.2 (Matrix Science, Boston, MA). MS/MS search parameters included fixed modifications (cysteine: Methylthio), variable modifications (asparagine, glutamine: deamidated; methionine: oxidation), and maximum one missed cleavage. Thermo *.raw files were converted into Mascot Generic Format using MSConvert in Protewizard. Files with the *.dat extension were exported from Mascot and loaded into Scaffold Q+ 4.4.1.1 (Proteome Software, Portland, OR) and used to calculate normalized spectral count for each protein from each sample. Scaffold protein and peptide thresholds were set at 95%, with a minimum peptide number of two per protein–this resulted in a protein false discovery rate (FDR) of 2.9% and a peptide FDR of less than 0.09% [27],[28]. The mass spectrometry proteomics data have been deposited to the ProteomeXchange Consortium [29] via the PRIDE partner repository [30] with the dataset identifiers PXD003096 and PXD003097.

### Metabolite mass spectrometry

Adult *D*. *citri* (50 from each population) were flash frozen, weighed, cryoground, and used for metabolite extraction. Metabolite extracts from CLas(+) and CLas(-) *D*. *citri* were prepared following a protocol based on the method used in the discovery of diaphorin [6]. Ground insects (~25 mg) were extracted three times in 500 μL methanol with shaking at 4°C. The three methanol extracts from each sample were combined, dried, and resuspended in 100 μLisopropanol. The integrated peak area results are normalized to account for differences in the starting mass of *D*. *citri* samples.

For each *D*. *citri* extract, 7 μl of sample was injected for analysis with HPLC-ESI-MS (5% acetonitrile for 3 min, 5–50% acetonitrile gradient for 40 min, 50–100% acetonitrile gradient for 10 min, 100% acetonitrile for 3 min, then returning to 5% acetonitrile for 6 minutes). Chromatography was performed with an Eclipse XDB-C18 column (5 μm resin, 4.6x250mm) with an Agilent 1100 series HPLC. Mass spectra were acquired on a Micromass Quattro II and analyzed using Masslynx v.4.1. The *m/z* range acquired was 200–700 *m/z* in positive ion mode, cone voltage was set to 35V, and source block temperature was 140°C.

## Supporting Information

S1 Fig *Profftella* quantitation in CLas(+)/(-) *D*. *citri* DNA samples. *Profftella* copy number was estimated by qPCR. Ct values from biological samples were compared to a dilution series of a synthetic plasmid corresponding to the *Profftella* 16s rRNA target gene. Natural logarithm of estimated *Profftella* copy number per insect equivalent is shown on the Y-axis. CLas(-): N = 4; CLas(+): N = 6 All biological samples were analyzed in triplicate, mean Ct value plus standard variation given for all replicates from each condition.
(TIF)

S1 Table Scaffold output of Mascot data from whole insect peptide mass spectra. Normalized peptide spectral count numbers for all proteins identified in all biological samples—three replicates each CLas(+) and CLas(-) *D*.*citri*. Fold difference: CLas(+) average/CLas(-) average.
(XLSX)

S2 Table Scaffold peptide report. Peptide sequences and protein IDs, and peptide and protein probability scores. Spectral count data for each peptide, including exclusive unique peptide count, exclusive unique spectrum count, and percentage protein sequence coverage.
(XLSX)

S3 Table *D*. *citri* endosymbiont proteins differentially expressed between CLas(+) and CLas(-) insects. Average normalized spectral count (N = 3) for protein samples from CLas(+) and CLas(-) adult *D*. *citri*. Fold difference CLas(+)/CLas(-). Thresholds for protein differential expression: T-test p-value< = 0.05, fold difference > = 2, < = 0.5.
(XLSX)

S4 Table *D*. *citri* proteins identified as up-regulated in CLas(+) insects. Average normalized spectral count (N = 3) for protein samples from CLas(+) and CLas(-) adult *D*. *citri*. Fold difference CLas(+)/CLas(-). Thresholds for protein differential expression: T-test p-value< = 0.05, fold difference > = 2.
(XLSX)

S5 Table *D*. *citri* proteins identified as down-regulated in CLas(+) insects. Average normalized spectral count (N = 3) for protein samples from CLas(+) and CLas(-) adult *D*. *citri*. Fold difference CLas(+)/CLas(-). Thresholds for protein differential expression: T-test p-value< = 0.05, fold difference < = 0.5.
(XLSX)

S6 Table D. citri and endosymbiont proteins identified as up-regulated in CLas(+) Percoll fractions. Average normalized spectral count (N = 3) for protein samples from CLas(+) and CLas(-) D. citri Percoll fractions. Fold difference CLas(+)/CLas(-). Thresholds for protein differential expression: Ttest p-value< = 0.05, fold difference > = 2.
(XLSX)

S7 Table D. citri proteins identified as down-regulated in CLas(+) Percoll fractions. Average normalized spectral count (N = 3) for protein samples from CLas(+) and CLas(-) D. citri Percoll fractions. Fold difference CLas(+)/CLas(-). Thresholds for protein differential expression: Ttest p-value< = 0.05, fold difference > = 2.
(XLSX)

S8 Table Scaffold output of mascot data from Percoll fraction peptide mass spectra. Normalized peptide spectral count numbers for all proteins identified in Percoll fraction samples. Fold difference: CLas(+) average/CLas(-) average.
(XLSX)

S9 Table Scaffold peptide report for Percoll fraction analysis. Peptide sequences and protein IDs, and peptide and protein probability scores. Spectral count data for each peptide, including exclusive unique peptide count, exclusive unique spectrum count, and percentage protein sequence coverage.
(XLSX)
