# Supplementary figures and images for: Metabolic Interplay between the Asian Citrus Psyllid and Its Profftella Symbiont: An Achilles’ Heel of the Citrus Greening Insect Vector
Source: PLoS One. 2015 Nov 18;10(11):e0140826. doi: 10.1371/journal.pone.0140826 (PMC4651294; doi:10.1371/journal.pone.0140826)

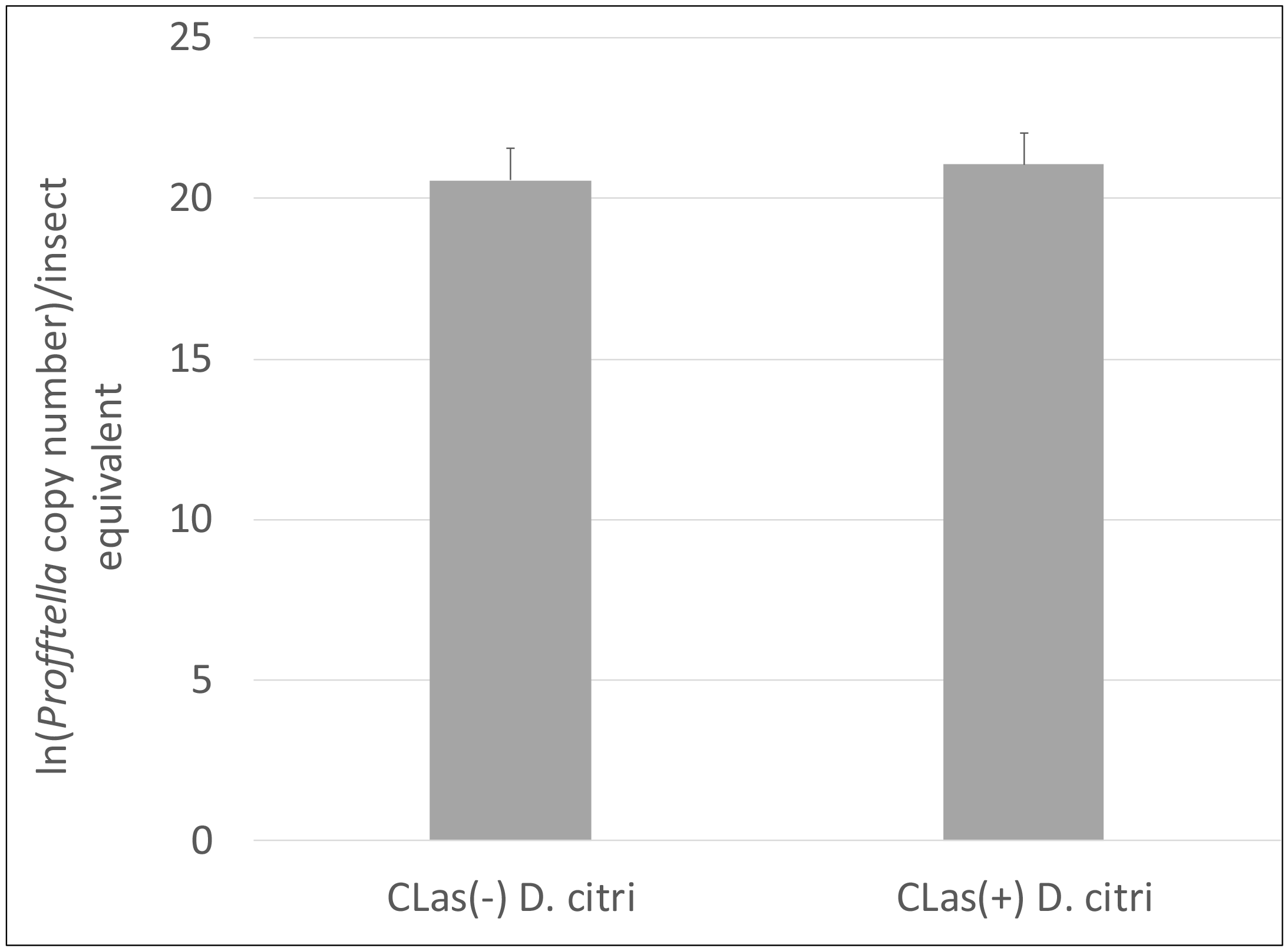

Supplement: S1 Fig — Profftella copy number was estimated by qPCR. Ct values from biological samples were compared to a dilution series of a synthetic plasmid corresponding to the Profftella 16s rRNA target gene. Natural logarithm of estimated Profftella copy number per insect equivalent is shown on the Y-axis. CLas(-): N = 4; CLas(+): N = 6 All biological samples were analyzed in triplicate, mean Ct value plus standard variation given for all replicates from each condition. (TIF) [file pone.0140826.s001.tif]
